# Postharvest Application of 24-Epibrassinolide Reduces Chilling Injury Symptoms and Enhances Bioactive Compounds Content and Antioxidant Activity of Blood Orange Fruit

**DOI:** 10.3389/fpls.2021.629733

**Published:** 2021-02-11

**Authors:** Fariborz Habibi, María Serrano, Lorenzo Zacarías, Daniel Valero, Fabián Guillén

**Affiliations:** ^1^Department of Horticultural Science, School of Agriculture, Shiraz University, Shiraz, Iran; ^2^Department of Agro-Food Technology, Miguel Hernández University of Elche, Orihuela, Spain; ^3^Department of Applied Biology, Miguel Hernández University of Elche, Orihuela, Spain; ^4^IATA, Consejo Superior de Investigaciones Científicas (CSIC), Paterna, Spain

**Keywords:** physiological disorder, sugar, organic acids, anthocyanins, polyphenols, ion leakage

## Abstract

Blood oranges (*Citrus sinensis* L. Osbeck cv. Sanguinello) fruit were treated with 24-epibrassinolide (Br) at 1, 5, and 10 μM previous to storage at 5°C during 42 days. The samples were analyzed after 14, 28, and 42 days plus 2 days at 20°C. Chilling injury was reduced in Br-treated fruit based on the lower percentage of electrolyte leakage and visual symptoms of peel dehydration and browning. Treated fruit showed lower acidity losses, due to retention of the main organic acids’ concentration (citric and malic acids), as well as was higher content of sugars (sucrose, fructose, and glucose), especially in those fruit treated with the highest concentration (10 μM). Total phenolics and hydrophilic total antioxidant activity (H-TAA) decreased in control fruit over storage, while Br-treated fruit showed significantly higher concentration. In addition, total anthocyanins were enhanced in Br-treated oranges, which were correlated with color Hue angle. Overall, the application of Br at 10 μM provides results increasing the storability of blood oranges and their content on bioactive compounds with antioxidant activity.

## Introduction

Spain is the sixth world producer of oranges with 3,500 Mt but the first exporter worldwide, most of them being sweet oranges although only 1% are blood oranges with an estimated area of only 1,500 ha (MAPAMA, 2019). Blood oranges are constituted by three main cultivars, “Moro,” “Tarocco,” and “Sanguinello.” The “Sanguinello,” also called “Sanguinelli” in the United States, was discovered in Spain in 1929, as a spontaneous mutation and has reddish skin, few seeds, and sweet and tender flesh. Consuming oranges, particularly blood oranges, have gained increased interest due to their high phenolic compound and anthocyanin content, and thus the literature describing the chemical composition of blood oranges and their juice has grown in recent decades (Fallico et al., 2017).

Two main anthocyanins have been identified in blood oranges, cyanidin 3-glucoside and cyanidin 3-(6″-malonylglucoside), although their contents are highly dependent on genotype, maturity, and environmental growth conditions (Lo Piero, 2015). It is known that anthocyanin content in blood oranges is low temperature-dependent and its biosynthesis stimulated during day/night changes (Butelli et al., 2012). Similarly, anthocyanin content in blood orange fruit may increase drastically through cold postharvest storage for a few weeks (Lo Piero et al., 2005).

Low-temperature storage is a commonly used practice to extend the commercial life of fresh citrus fruit, and it is required as a quarantine treatment for citrus export to different countries (El-Otmani et al., 2011; Zacarías et al., 2020). However, storage of blood oranges at temperatures below 8°C may cause chilling injury (CI) and alteration in their organoleptic and external properties (Rapisarda et al., 2001). The major CI symptoms in blood oranges and other citrus fruit are pitting and necrotic areas in the flavedo (external clouted layer of the skin) and changes in cell membrane integrity, which may affect cell structure and stability, and then provoking the loss of compartmentalization and the increase of electrolyte leakage (EL) (Lado et al., 2016). Chilling stress can be decreased the fruit quality of blood oranges during cold storage (Habibi et al., 2020b).

Oxidative stress appears to be one of the most common consequences of CI in plants, which, in general, have developed an enzymatic and non-enzymatic antioxidant system as the first defense barrier from oxidative stress and the damaging effects of reactive oxygen species (ROS) (Sharma et al., 2020). The antioxidant defense systems consist of secondary metabolites, such as polyphenol compounds, carotenoids, among others, and enhanced activity of antioxidant enzymes, as has been observed in blood oranges (Habibi et al., 2019). In recent years, several innovative compounds have been used to alleviate CI when they are applied as postharvest treatments (Valero and Serrano, 2010; Sharma et al., 2020). Particularly in blood oranges, γ-aminobutyric acid (GABA), methyl jasmonate (MeJA), or methyl salicylate (MeSA) have been shown to reduce CI symptoms of “Moro” blood oranges, that were mainly manifested as reduction of EL, malondialdehyde (MDA), and hydrogen peroxide content and enhanced the activity of the antioxidant enzymes (Habibi et al., 2019). These treatments were also effective in enhancing total phenolics, and total anthocyanin content, as well as the major individual anthocyanin concentration, cyanidin 3-glucoside, and cyanidin 3-(6″-malonylglucoside), with respect to control during the whole cold storage period (Habibi et al., 2020b).

Brassinosteroids (Brs), a group of plant hormones, are occurring universally in plant tissues and involved in a wide range of physiological processes, including plant growth, floral initiation, pathogen infection, and ethylene synthesis (Aghdam et al., 2016; Wei and Li, 2020). Several Br compounds have been identified so far, and although C_28_ Brs are the most ubiquitous in nature, the 24-epibrassinolide (Br) is the most used in modern agriculture (Hayat and Ahmad, 2011; Bajguz et al., 2020).

In climacteric fruit, in which the ripening process is regulated by the plant hormone ethylene, the potential of Brs for the regulation of fruit ripening has also been investigated. Thus, Zhu T. et al. (2015) reported that Br treatment in tomato increases ethylene production and the expression of ethylene biosynthesis genes during ripening. Moreover, exogenous Br application accelerated fruit ripening that was accompanied by increases in lycopene and carbohydrate levels (Vardhini and Rao, 2002). In non-climacteric fruit, such as grape berries, exogenous Br application substantially enhanced total soluble solids and total acidity as well as increased antioxidants, phenolics, and ascorbic acid content (Asghari and Rezaei-Rad, 2018). In mandarin fruit, treatment with Brs reduced weight loss and increased proline and the sugars D-xylose and D-galactose (Zhu F. et al., 2015). In fruit exposed to low temperature, postharvest Br treatment has been found to be beneficial in alleviating chilling stress, as reported for pepper (Wang et al., 2012), tomato (Aghdam et al., 2012), and peach (Gao et al., 2016) by reducing the EL (Li et al., 2012). In citrus fruit, the application of Brs led to alleviation of CI in “Washington navel” oranges during storage at 3°C (Ghorbani and Pakkish, 2014). In the same way, Br in combination with hot water treatment significantly reduced CI symptoms due to lower oxidative damage in lime fruit stored at 5°C (Rezakhani and Pakkish, 2017).

However, as far as we know, the role of Brs in the postharvest performance of blood oranges has not been investigated. Thus, the aim of this work was to study the effect of postharvest application of Br (at 1, 5, and 10 μM) on chilling damage, organoleptic quality (sugars and organic acids), the content of bioactive compounds (ascorbic acid, total phenolics, and total anthocyanins), and antioxidant activity, measured from both hydrophilic and lipophilic extracts during postharvest cold storage (5°C) of blood orange fruit.

## Materials and Methods

### Plant Material and Experimental Design

Blood oranges (*Citrus sinensis* L. Osbeck cv. Sanguinello) were harvested at random from adult trees grown under standard conditions in a commercial orchard located in Valencia, and immediately transported to the Postharvest laboratory of Miguel Hernández University of Elche (Orihuela, Spain) under refrigerated conditions. Fruit uniform in size and color, and free of damage or external defects were selected and divided into four lots of 45 blood oranges (three replicates of five fruit) for storage experiments and treatments. Similarly, 15 fruit were used immediately after harvest to analyze fruit properties at harvest (0 day of storage).

24-Epibrassinolide (Br) was purchased from Sigma (Sigma-Aldrich Co., Madrid, Spain) and aqueous solutions of Br was prepared by dissolving them in a small amount of ethanol and bringing to the final volume of 10 L with water at concentration of 0 (control), 1, 5, and 10 μM. A surfactant Tween 20^®^ (Polyoxyethylenesorbitan monolaurate polyethylene glycol, Sigma-Aldrich Co., Madrid, Spain) at the rate of 0.1% was added to obtain better retention and penetration of Brs solution. Treatments were performed by immersion during 10 min and fruit left to dry at room temperature for 2 h until stored in the cold chambers at 5°C and 90% RH for 14, 28, and 42 days. For each sampling date and treatment, three lots of five fruit were sampled at random and kept 2 days at 20°C (shelf-life) and then the following parameters were measured.

### Evaluation of Physicochemical Properties

Weight loss was determined individually in each fruit by measuring the eight of the fruit at intervals and calculating the percentage of weight loss respect to the initial value, and data were expressed as mean ± SE. Color was measured on the peel (external) and on the pulp (internal), and for both was determined along with six points of the equatorial perimeter in each fruit, using the CIE Lab system in a colorimeter (CRC200, Minolta Camera Co., Tokyo, Japan). After recording L^∗^, a^∗^, and b^∗^ parameters, color was expressed as Hue angle (arctan b^∗^/a^∗^). After color determination, fruit were sliced in two halves and the juice from each triplicate was mixed to obtain a homogeneous sample and titratable acidity (TA) was determined in duplicate by automatic titration (785 DMP Titrino, Metrohm) with 0.1 N NaOH up to pH 8.1, using 1 mL of diluted juice in 25 mL distilled H_2_O, and results (mean ± SE) were expressed as g kg^–1^ citric acid equivalent.

### Individual Sugars and Organic Acids Content

Sugars and organic acids were determined following the protocol by Serrano et al. (2009) with minor modifications. From the juice obtained from each replicate, 1 mL was filtered through 0.45 μm Millipore filter and injected into a high-performance liquid chromatography (HPLC) system (Hewlett-Packard HPLC series 1100). The mobile phase was 0.1% phosphoric acid and samples were run under isocratic conditions at a flow rate of 0.5 mL min^–1^. Sugars and organic acids were separated by using a Supelco column (Supelcogel C-610H, 30 cm 7.8 mm, Supelco Park, Bellefonte, PA, United States), and total time chromatogram was 45 min. Organic acids were detected by absorbance at 210 nm and sugars by refractive index detector. Results (mean ± SE) were expressed as g kg^–1^. A standard curve of pure sugars (sucrose, glucose, and fructose) and organic acids (citric, malic, ascorbic, succinic, and fumaric acids) purchased from Sigma (Sigma-Aldrich Co., Madrid, Spain) was used for quantification. Results are the mean ± SE of three replicates.

### Electrolyte Leakage (EL)

For EL evaluation, ten disks (0.5 cm diameter) of the fruit peel were excised using a cork borer and rinsed twice with distilled water. Peel disks were inserted in a 15 mL falcon tube with 10 mL of distilled water and shaken at room temperature for 3 h. First electrolyte leakage (EC1) and second electrolyte leakage (EC2) were measured after 3 h of shaking and after autoclave at 121°C for 20 min and cooling down at room temperature, respectively, using a conductivity-meter (AZ-86505, Taiwan). The percentage of EL was calculated using the following formula (Wang et al., 2014). Two measurements were performed for each replicate and the results are the mean ± SE.

$$\mathrm{EL}\left(\%\right)=\frac{\mathrm{EC1}}{\mathrm{EC2}}\times 100$$

### Bioactive Compounds and Antioxidant Activity

Total phenolics, total anthocyanins, and total antioxidant activity (TAA) from hydrophilic (H-TAA) and lipophilic (L-TAA) extracts were determined according to Serrano et al. (2009). Briefly, 10 mL from the juice obtained from each replicate were mixed with 10 mL water/methanol (2:8) containing 2 mmol L^–1^ sodium fluoride (NaF) which inactivates polyphenol oxidase activity, and thus preventing phenolic degradation. After centrifugation at 10,000 × *g* for 10 min at 4°C the supernatant was used to determine total phenol content using the Folin-Ciocalteu, and results (mean ± SE) were expressed as mg L^–1^ gallic acid (GA) equivalent.

To extract anthocyanins, 10 mL from the juice obtained from each replicate were mixed with 15 mL of methanol/formic acid/water (25:1:24, v/v/v) and then centrifuged at 10,000 × *g* for 10 min at 4°C. Total anthocyanin content was quantified using a spectrophotometer at 520 nm and expressed as mg L^−1^ of cyanidin 3-*O*-glucoside (cy 3-gluc.) equivalent, the cyanidin 3-glucoside molar absorption coefficient being 23,900 L cm^–1^ mol^–1^ and molecular weight of 449.2 g mol^–1^).

Total antioxidant activity was determined from hydrophilic (H-TAA) and lipophilic (L-TAA) compounds in the same extraction. In brief, for each replicate, 10 mL of the juice mixed with 10 mL of 50 mmol L^–1^ phosphate buffer pH 7.8 and 5 mL of ethyl acetate, and then centrifuged at 10,000 × *g* for 15 min at 4°C. The upper phase contained the lipophilic compounds while the lower phase the hydrophilic ones. For both, TAA was determined by using the ABTS enzymatic system, and the decrease in absorbance was recorded at 730 nm. Results (mean ± SE) were expressed as mg L^–1^ Trolox equivalents.

### Statistical Analysis

The experiment was conducted according to a completely randomized design and with 3 replicates in two-factors (treatment and storage time). Data were analyzed for variance by using SPSS software package (Version 21, SPSS Institute Inc., United States). When interactions between treatments were significant (*P* < 0.05), the effect of each treatment was determined by separating the means by Least Significant Difference (LSD). Linear regressions were performed between Br applied concentration and each analyzed parameter for each storage sampling date using Sigma Plot software package V. 11.

## Results

### Physicochemical Properties

As expected, cumulative weight loss increased during storage for both control and Br-treated fruit (Table 1), although the final value was affected by Br treatments. Thus, at the end of storage period, the lowest weight loss was attained in blood oranges treated with Br 10 μM (9.01 ± 0.43%) which was statistically different with respect to the value of untreated fruit (11.14 ± 0.48%). The linear regression revealed that the reduction of percentage of weight loss was positively correlated (*R*^2^ = 0.991) with increasing the Br concentration. Titratable total acidity (TA) decreased during storage of control fruit and it was significantly affected by treatment (Table 1). The highest TA retention was obtained in those fruit treated with Br 10 μM, since the initial levels did not significantly change (*P* < 0.05) after 42 days of storage. In control blood oranges the reduction of TA was significant (*P* < 0.05) and after 42 days of storage initial TA was reduced by 43.2%. Similar to weight loss, the linear regression revealed a negative correlation between TA reduction (*R*^2^ = −0.775) with increasing Br concentrations.

**TABLE 1:** Effect of brassinosteroid (Br) treatment on physicochemical characteristics of blood oranges during storage at 5°C.*

Treatment	Days	Weight loss (%)	Total acidity (TA) g kg^–1^	External Hue	Internal Hue
Control	Harvest	–	23.6 ± 0.5a	54.45 ± 1.21a	66.11 ± 1.49a
	14	6.25 ± 0.45aA	20.1 ± 0.3bA	43.32 ± 1.46bA	65.78 ± 1.45aA
	28	9.33 ± 0.43bA	17.5 ± 0.4cA	43.57 ± 1.18bA	63.51 ± 1.41bA
	42	11.14 ± 0.48cA	13.4 ± 0.5dA	38.37 ± 1.64cA	62.09 ± 1.39bA
Br 1 μM	14	5.96 ± 0.07aA	22.8 ± 0.9aB	52.77 ± 1.21aB	62.36 ± 1.13bB
	28	7.94 ± 0.95bB	20.8 ± 0.5bB	48.96 ± 1.32bB	60.13 ± 1.28bB
	42	10.83 ± 0.27cAB	18.7 ± 0.4cB	47.77 ± 1.24bB	59.71 ± 1.75bA
Br 5 μM	14	5.51 ± 0.23aAB	22.8 ± 0.4aB	55.48 ± 0.88aB	64.14 ± 1.44aA
	28	7.19 ± 0.22bB	19.7 ± 0.4bB	50.96 ± 1.32aB	63.13 ± 1.81aA
	42	9.97 ± 0.23cB	17.4 ± 0.5cB	47.77 ± 1.24bB	60.35 ± 1.15bA
Br 10 μM	14	5.22 ± 0.24aB	23.2 ± 0.2aB	56.18 ± 0.63aB	60.11 ± 1.22bB
	28	7.14 ± 0.40bB	22.9 ± 0.7aC	50.27 ± 1.11bB	56.85 ± 1.47cC
	42	9.01 ± 0.43cB	21.3 ± 0.9aC	50.01 ± 1.24bB	50.01 ± 1.24dB

* For each treatment, mean followed by different small letter denotes significant difference at P < 0.05 along storage. For each sampling date, mean followed by different capital letter denotes significant difference at P < 0.05 among treatments.

**FIGURE 1:**
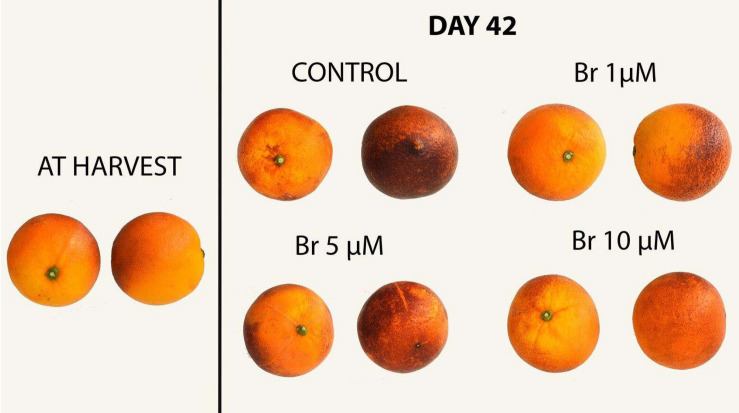
External appearance of blood oranges at harvest and after 42 days of storage.

Fruit color was measured in both external (peel color) and internal (segments) sections, and is expressed as Hue angle. For both tissues, Hue angle decreased during storage and the final value was also affected by Br treatment (Table 1). The linear regression revealed that the reduction of Hue angle of the pulp color was negatively correlated (*R*^2^ = −0.845) with increasing Br concentrations, and thus fruit treated with Br 10 μM showed significantly higher internal color after prolonged cold storage. Figure 1 shows the visual aspect of blood oranges at harvest and after 42 days of storage.

### Individual Sugars and Organic Acids

Analysis of the individual organic acids’ concentration revealed that the major ones were citric > malic > ascorbic > succinic > oxalic acid (Table 2). During storage, acid concentration in control fruit experienced a significant reduction (*P* < 0.05), between 32 and 35% in citric, malic and succinic, and 70% in ascorbic after harvest to 42 days, respectively. However, the effect of Br treatments is maintained significantly higher the content of citric acid during storage, especially for the highest concentration (10 μM), which did not change during storage. This Br dose was also the most effective in avoiding the reduction of the concentration of the other organic acids. Interestingly, the concentration of ascorbic acid remained unchanged in blood oranges treated with Br at 10 μM, while control samples showed a significant drop during cold storage. The linear regression revealed that the citric and ascorbic acid retention was positively correlated (*R*^2^ = 0.845 and *R*^2^ = 0.934, respectively) with increasing Br concentrations.

**TABLE 2:** Effect of brassinosteroid (Br) treatment on individual content of organic acids (g kg^–1^) of blood oranges during storage at 5°C.*

Treatment	Days	Citric acid	Malic acid	Ascorbic acid	Succinic acid	Oxalic acid
Control	Harvest	20.31 ± 0.98a	1.15 ± 0.17a	0.51 ± 0.04a	0.48 ± 0.04a	0.05 ± 0.01a
	14	18.27 ± 1.06bA	1.11 ± 0.05aA	0.38 ± 0.03bA	0.40 ± 0.09aA	0.05 ± 0.01aA
	28	16.88 ± 0.29cA	1.05 ± 0.11aA	0.31 ± 0.07bA	0.40 ± 0.06aA	0.04 ± 0.01aA
	42	13.72 ± 0.57dA	0.78 ± 0.07bA	0.15 ± 0.07cA	0.31 ± 0.02bA	0.03 ± 0.02aA
Br 1 μM	14	19.27 ± 0.12aA	1.09 ± 0.04aA	0.37 ± 0.01bA	0.51 ± 0.01aA	0.04 ± 0.01aA
	28	17.82 ± 0.46bB	1.18 ± 0.06aA	0.34 ± 0.03bA	0.42 ± 0.07aA	0.04 ± 0.01aA
	42	14.79 ± 0.78cB	0.87 ± 0.11bA	0.19 ± 0.06cA	0.37 ± 0.04bA	0.05 ± 0.01aA
Br 5 μM	14	18.51 ± 0.42bA	1.25 ± 0.13aA	0.44 ± 0.11aA	0.48 ± 0.07aA	0.04 ± 0.01aA
	28	17.56 ± 0.99bB	1.07 ± 0.02aA	0.38 ± 0.12bA	0.44 ± 0.05aA	0.05 ± 0.01aA
	42	15.25 ± 0.63cA	0.94 ± 0.05aA	0.27 ± 0.07cB	0.41 ± 0.02aAB	0.04 ± 0.01aA
Br 10 μM	14	21.71 ± 0.97aB	1.28 ± 0.12aA	0.52 ± 0.06aB	0.55 ± 0.07aB	0.04 ± 0.01aA
	28	19.68 ± 0.12aC	1.27 ± 0.11aB	0.47 ± 0.08aB	0.51 ± 0.04aB	0.05 ± 0.01aA
	42	19.29 ± 0.11aB	1.03 ± 0.09aB	0.42 ± 0.09aC	0.48 ± 0.02aB	0.05 ± 0.01aA

* For each treatment, mean followed by different small letter denotes significant difference at P < 0.05 along storage. For each sampling date, mean followed by different capital letter denotes significant difference at P < 0.05 among treatments.

**FIGURE 2:**
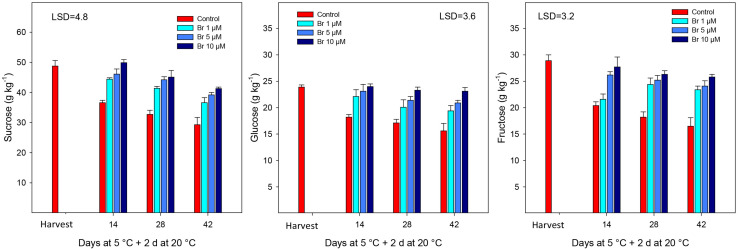
Concentration of sugars (g kg^–1^) from control and Br-treated fruit during storage. Data are the mean ± SE. LSD denotes the level of significance during storage and among treatments at *P* < 0.05.

With respect to individual sugars, sucrose was the major followed by fructose and glucose (Figure 2). All sugars showed a similar behavior during storage, which was a significant (*P* < 0.05) reduction in the concentration in control fruit during storage, which was significantly avoided in Br-treated blood oranges. When linear regression was performed, the loss of individual sugars was negatively correlated with increasing Br concentrations; with *R*^2^ = −0.921 for sucrose, −0.906 for fructose, and −0.833 for glucose.

### Electrolyte Leakage (EL)

**FIGURE 3:**
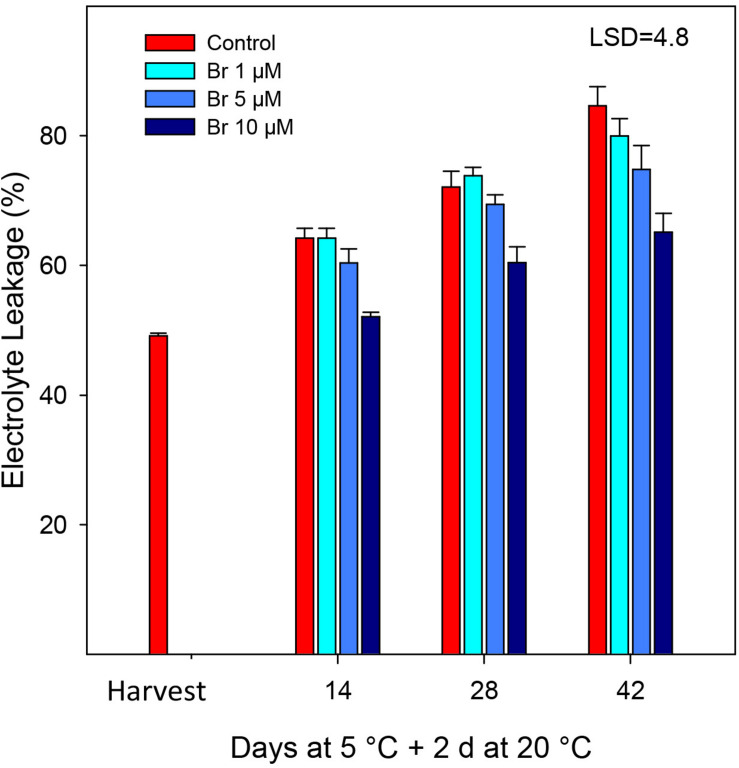
Percentage of electrolyte leakage (EL) from control and Br-treated fruit during storage. Data are the mean ± SE. LSD denotes the level of significance during storage and among treatments at *P* < 0.05.

The percentage of EL increased for most of the treatments during storage at 5°C plus 2 days at 20°C (Figure 3). However, control fruit exhibited a progressive and significantly (*P* < 0.05) increase in EL during storage, which was near 60% higher than the initial after 42 days. Application of 10 μM was the only treatment that significantly reduced (*P* < 0.05) EL with respect to non-treated fruit, that it was manifested as early as 2 weeks after storage (Figure 3).

### Bioactive Compounds and Antioxidant Activity

**FIGURE 4:**
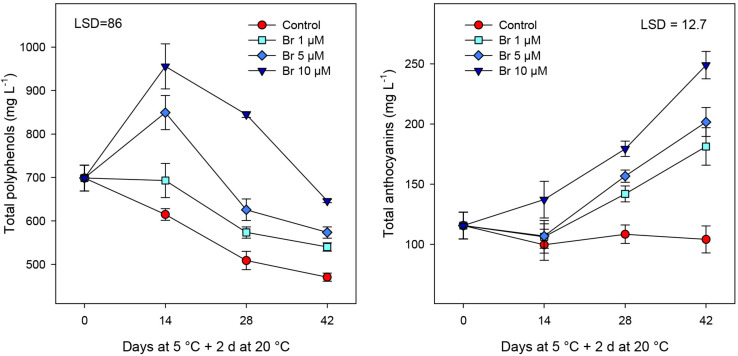
Concentration of total phenolics and total anthocyanins (mg L^–1^) from control and Br-treated fruit during storage. Data are the mean ± SE. LSD denotes the level of significance during storage and among treatments at *P* < 0.05.

The concentration of bioactive compounds and total phenolics showed a progressive reduction during storage in non-treated blood oranges (Figure 4). In fruit treated with Br at 5 and 10 μM, total phenolics significant increase at 14 days, declining thereafter but they remained higher than in control fruit at the end of storage period. On the contrary, the concentration of anthocyanins significantly (*P* < 0.05) increased in all Br-treated blood oranges, although reaching higher content in fruit treated with the highest Br concentration (Figure 4). In addition, there was a close relationship between retention of total phenolics (*R*^2^ = 0.975) and total anthocyanins (*R*^2^ = 0.989) content and the Br dose, being 10 μM the most effective.

**FIGURE 5:**
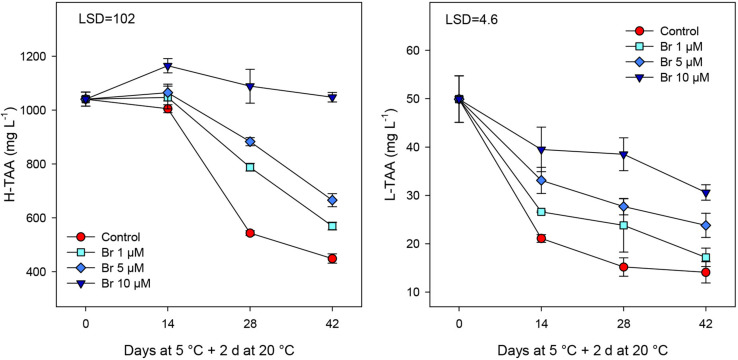
Total antioxidant activity (mg L^–1^ Trolox equivalent) in hydrophilic (H-TAA) and lipophilic (L-TAA) extracts from control and Br-treated fruit during storage. Data are the mean ± SE. LSD denotes the level of significance during storage and among treatments at *P* < 0.05.

Total antioxidant activity (TAA) was measured from hydrophilic (H-TAA) and lipophilic (L-TAA) extracts, and for both parameters a significant (*P* < 0.05) reduction was observed during cold storage of control fruit (Figure 5). Br treatment significantly (*P* < 0.05) reduced the decline in both antioxidant activities, being the effect dose-dependent, with a regression coefficient of 0.994 for H-TAA and 0.941 for L-TAA Thus, the highest concentration was found for blood oranges treated with Br at 10 μM, since remained unchanged for H-TAA and a value of 30.6 ± 1.6 for L-TAA.

## Discussion

Blood oranges, like other citrus fruit, are very prone to develop postharvest physiological disorders after prolonged storage periods (Zacarías et al., 2020). At ambient temperatures, blood oranges increase respiration rate, transpiration and fungal decay, accelerate senescence, and affecting the content of bioactive compounds, all of them resulting in a significant reduction of fruit quality and shelf-life period (Rapisarda et al., 2001). Despite cold storage is probably the most effective to maintaining fruit quality and prolong the postharvest life, fruit of many citrus cultivars are sensitive to develop chilling injury (CI) of the peel, that significant downgrade external fruit quality and many originate rejection in the fresh-fruit markets. The classical symptoms of CI are pitting of the peel, superficial scald-like symptoms, and peel browning (Zacarías et al., 2020). Recently, several postharvest treatments with natural-occurring elicitors have been effective in reducing blood orange CI symptoms, such as GABA, MeJA, or MeSA, and in turn prolonged the storability (Habibi et al., 2019). As an alternative, we have used for the first time a postharvest application of Br at three concentrations (1, 5, and 10 μM) on blood oranges before storage at 5°C. Br at the concentration of 10 μM was the most effective on maintaining the physicochemical parameters, such as titratable acidity (TA), individual sugars and organic acids, and reduced the percentage of weight loss (Tables 1, 2 and Figure 2).

As primary metabolites and energy sources, sugars play important roles in the fruit development and quality since they accumulate to very high levels in blood oranges during ripening. However, during postharvest storage, sugars and organic acids tend to decrease, mostly because of the use of these compounds as respiratory substrates, as well as for the synthesis of new molecules (Valero and Serrano, 2010). However, the application of Br had a positive effect avoiding sugar decline and then maintaining higher concentration (sucrose, glucose, and fructose) during the whole storage period. A similar effect was also observed for the decline in acids, mainly citric and malic acids, that were retained at a higher concentration at the highest Br dose (10 μM). The relationship between Br and sugars has been poorly studied, and most of the evidences are referred to grape (both in wine and table cultivars) during growth and developmental cycle and the increase in crop yield (at preharvest levels). Thus, Xu et al. (2015) demonstrated that Br enhanced sugar unloading in grape berries at veraison stage due to a significant promotion of the invertase (both acid and neutral) and sucrose synthase activities at various stages of ripening. “Thompson” seedless table grapes were sprayed with Br (at 0.3 and/or 6 μmol L^–1^) at three different stages, and treated berries had a high content of both sugars and organic acids (Asghari and Rezaei-Rad, 2018), although they did not find a significant difference between the two Br concentrations assayed. In this sense, application of Br it is likely that may reduce respiration rate, since both sugars and organic acids are the primary substrates for fruit respiration, and would explain the reduction in weight loss (Table 1), since the products of respiration are CO_2_ and H_2_O vapor (Valero and Serrano, 2010).

Electrolyte leakage (EL) is considered a good indicator of cell membrane and permeability, and often it has been related to the sensitivity of fruit to low temperatures and the incidence of chilling symptoms (Wang et al., 2014). Postharvest application of Br significantly reduced EL compared with control fruit, the 10 μM Br being the most effective. In blood oranges, the occurrence of CI symptoms is the result of a series of physiological and biochemical responses to cold temperature, manifested as external pitting (Lafuente and Zacarías, 2006; Lafuente et al., 2017; Habibi et al., 2019). Figure 1 shows the visual aspect of the blood oranges after 42 days of storage, at which the higher CI symptoms (peel pitting and browning) were clearly observed in control fruit. Peel browning was also associated with an increasing rate of water loss that was also reduced in Br-treated fruit. These results agree with those obtained in “Washington navel” oranges, in which 0.75 and 1.5 ppm of Brs effectively reduced CI (Ghorbani and Pakkish, 2014). Similarly, treatment of eggplant with 10 μmol L^–1^ Br reduced EL that in turn reduced the symptoms of CI at 1°C, mainly pulp browning (Gao et al., 2015). In climacteric fruit such as peach and tomato, the immersion in a 15 mM 24-epibrassinolide (Gao et al., 2016) or 6 mM (Aghdam and Mohammadkhani, 2014) significantly alleviated CI, which was manifested by reduced EL and malondialdehyde, and increased proline content.

Red-fleshed cultivars of sweet oranges are known as “blood oranges” that are highly appreciated in the market and by the consumer by their health-related properties (Fallico et al., 2017). Blood oranges differed from other sweet ones by their accumulation of anthocyanins, which confer a superior antioxidant activity of the juice (Lo Piero, 2015). Thereafter, other compounds that contribute to the health-promoting properties of blood oranges include other phenolics and L-ascorbic acid (vitamin C) content, which account for about 70% of their total antioxidant activity (Arena et al., 2001; Lo Piero et al., 2014). In addition, antioxidant enzymes including superoxide dismutase (SOD), catalase (CAT), and ascorbate peroxidase (APX) can influence flesh antioxidant activity but they could not be responsible for the enhancing antioxidant system in blood oranges during cold storage (Habibi et al., 2020b). In the current work, Br treatments, especially 10 μM, maintained higher levels of total phenolics, total antioxidant activity (both H-TAA and L-TAA) and ascorbic acid, and increased total anthocyanins during storage at 5°C plus 2 days at 20°C (Figures 4–5 and Table 2). These effects are noteworthy since cold storage of control fruit provoked reductions of 50–90% in different bioactive compounds and the antioxidant activity, then Br-treated fruit contained high levels of bioactive compounds and increased health-related properties. This higher content of phenolic compounds, and anthocyanins, besides to the antioxidant activity and the health-related attributes are components of crop defense systems against different biotic and abiotic stresses including CI (Tomás-Barberán and Espín, 2001). Similarly, postharvest application of Br at 15 μM in peach induced chilling tolerance and also stimulated phenolic and proline biosynthesis as well as to inhibition of oxidative stress (Gao et al., 2016). In this sense, hesperidin could be responsible for the induction of tolerance to CI, since it is the most abundant flavonoid in blood oranges (Cebadera-Miranda et al., 2019). Accumulation of chilling-induced phenolic compounds is usually considered as an adaptive mechanism to prevent cellular oxidative damage in plants (Rivero et al., 2001). Moreover, there is evidence confirming that phenolic compounds are able to detoxify ROS by donating hydrogen and decomposing peroxides (Rice-Evans et al., 1996). ROS accumulation CI-damaged fruit is a consequence of an imbalance between the generation and detoxification of ROS, which leads to damage to the cellular components and cellular membranes (Huang et al., 2008; Wang et al., 2014; Habibi et al., 2019). Brs have been reported to induce the activity of antioxidant enzymes that contribute to the suppression of ROS that accompanies the reduction of CI in several fruits, such as bell pepper (Wang et al., 2012), eggplant fruit (Gao et al., 2015), and “Washington navel” orange (Ghorbani and Pakkish, 2014). Then, it is likely that Brs may operate in blood oranges by a similar mechanism, by increasing the antioxidant systems that counteract the generation of ROS and would protect the fruit against chilling-induced damage.

It is also noteworthy that Br-treatments, especially at the concentration of 10 μM, maintained high levels of ascorbic acid (Table 2). The maintenance of ascorbic acid content by Br-treatments, especially at the concentration of 10 μM, is very important due to is an essential nutrient having an antioxidant role as vitamin C, with a final concentration of 0.48 g kg^–1^ (Table 2). The Regulation (EU) No 1169/2011, related to consumer information on food labeling, indicates a nutrient reference value (NRV) for daily vitamin C of 80 mg. According to this regulation, the consumption of 100 g of blood oranges pulp would cover at least 60% of these recommendations. Br at the concentration of 10 μM retained the losses found in control fruit for H-TAA, and this parameter has been associated with total phenolics and ascorbic acid since both are hydrophilic compounds. On the other hand, L-TAA is due to lipophilic compounds and in the case of blood oranges, these are tocopherol or vitamin E and carotenoids, mainly β-carotene and absence of lycopene (Cebadera-Miranda et al., 2019).

Appearance, including fruit color, is generally used as a selection criterion throughout the supply and consumer chain. The intensity of the pigmentation depends on several variables such as cultivar, soil type, climate, and environmental factors during growth and ripening. In addition, storage conditions have an impact on the color of blood oranges. For instance, superatmospheric storage enriched with O_2_ increased almost 10-times the content of anthocyanins compared with a normal atmosphere (Molinu et al., 2016). In control fruit, the external color (peel) measured as Hue angle, experienced a decline during storage, while in all Brs-treated fruit the value of Hue angle was significantly higher, especially of the 10 μM concentration (Table 1 and Figure 1). The significance of the decrease in Hue angle was a darker color of the peel due to the incidence of CI and browning, while the opposite is shown for the blood oranges treated with 10 μM Br. Regarding the internal color (pulp), Hue angle of control fruit did not change during storage, while blood oranges treated with 10 μM Br showed a significant reduction. The results of pulp color were confirmed with the measurements of total anthocyanins measured by spectrophotometer. In fact, correlation analysis shows that Hue color parameter was negatively correlated with total anthocyanins (*R*^2^ = −0.915). According to Lo Piero et al. (2005) and Butelli et al. (2012), anthocyanin accumulation in the flesh of blood oranges can be promoted by keeping the fruit at temperatures between 4 and 10°C, but the present results show that postharvest application of Br induced higher accumulation of anthocyanins, especially for the 10 μM concentration. There is no available literature regarding the effects of Br treatments on blood orange for comparative purposes, but the preharvest Br treatments on table and wine grapes, stimulated the accumulation of anthocyanins in both peel and pulp (Xu et al., 2015; Asghari and Rezaei-Rad, 2018).

Anthocyanin accumulation depends on storage temperatures and cultivars (Habibi et al., 2020a). It has been reported that blood orange cultivars are cold dependent for the synthesis of anthocyanin after harvest, but enhancement of anthocyanin at moderate temperature was significantly higher than cold temperature and efficiency of colder temperature (below 3°C) was inefficient (Habibi et al., 2020a). In addition, our previous study revealed that untreated blood oranges fruit need to store up to 90 days at a moderately cold temperature to reach a good internal red pigment (Habibi et al., 2020a). In addition, untreated fruit (control samples) stored for 42 days at 5°C. Therefore, this storage period was not enough for enhancing anthocyanin in untreated fruits.

In recent decades, postharvest treatment with some elicitors enhanced the chilling tolerance of blood oranges by some involved mechanisms (Habibi et al., 2019). In addition, elicitors can maintain cellular energy status and delay fruit senescence and leading to the use of energy for cellular metabolisms. For example, postharvest application of some elicitors such as putrescine has been increased total anthocyanin in “Moro” and “Tarocco” cultivars during 60 days of cold storage (Habibi and Ramezanian, 2017). Furthermore, postharvest treatment of some elicitors has been increased the phenylalanine ammonia-lyase (PAL) as reported in treated fruit with GABA, MeJA, and MeSA (Habibi et al., 2020b). These differences in anthocyanins accumulation are due to PAL activity. Therefore, postharvest application of elicitors could be an effective technique for increasing anthocyanin concentrations in blood orange fruit through cold temperature without CI symptom in treated fruit. In our study, treated fruit with Br had a lower CI and probably maintained the cellular energy status or increased PAL activity as reported in tomato fruit (Aghdam et al., 2012). Consequently, anthocyanin accumulation in Br-treated fruit was higher than in control samples. Stability and degradation of anthocyanin molecule depend on polyphenol oxidase (PPO) activity and PAL/PPO during cold storage as previously reported (Habibi et al., 2020b). Low activity of PPO activity is related to acidic conditions, higher content of citric acid, and ascorbic acid of blood oranges fruit as occurred in Br-treated fruit. In addition, CI in the control sample was higher than the treated fruit. It seems that all of these conditions affected anthocyanins accumulation in control samples. In addition, chilling-inducing temperatures (≤5°C) cannot enhance anthocyanin accumulation as previously reported in untreated blood oranges fruit (Carmona et al., 2017; Habibi et al., 2020a). Therefore, treated fruit had an enhancement of anthocyanin accumulation during 42 days at cold temperature with a significant difference with control samples.

## Conclusion

The findings of this study indicated that the application of Br at 10 μM was effective in reducing the incidence of CI and enhancing fruit quality during cold storage of blood oranges. The treatments have been shown to avoid the decline occurring in cold-stored non-treated fruit in many processes (color, taste, flavor, sugars, and organic acids). Then Br-treatment maintains the high color index and organoleptic quality (acidity, sugars, and organic acids), high bioactive compounds (phenolics, anthocyanins, and ascorbic acid), and hydrophilic and lipophilic total antioxidant activity, being the most effective concentration of 10 μM.

## Data Availability Statement

The raw data supporting the conclusions of this article will be made available by the authors, without undue reservation.

## Author Contributions

DV conceived and designed the work in association with other authors. FH, MS, FG, and LZ performed the investigations. FH performed most of the analytical determination in collaboration with the other authors. DV analyzed the data and wrote the manuscript. All authors approved the final version of the manuscript.

## Conflict of Interest

The authors declare that the research was conducted in the absence of any commercial or financial relationships that could be construed as a potential conflict of interest.

## References

[B1] Aghdam M. S. Mohammadkhani N. (2014). Enhancement of chilling stress tolerance of tomato fruit by postharvest brassinolide treatment. *Food Biol. Technol*. 7 909–914. 10.1007/s11947-013-1165-x

[B2] Aghdam M. S. Asghari M. Farmani B. Mohayeji M. Moradbeygi H. (2012). Impact of postharvest brassinosteroids treatment on PAL activity in tomato fruit in response to chilling stress. *Sci. Hortic*. 144 116–120. 10.1016/j.scienta.2012.07.008

[B3] Aghdam M. S. Babalar M. Sarcheshmeh A. A. (2016). “Impact of brassinosteroids on postharvest physiology of fruits and vegetables,” in *Eco-Friendly Technology for Postharvest Produce Quality*, ed. Siddiqui M. S. (London: Academic Press), 203–218. 10.1016/B978-0-12-804313-4.00006-2

[B4] Arena E. Fallico B. Maccarone E. (2001). Evaluation of antioxidant capacity of blood orange juices as influenced by constituents, concentration process and storage. *Food Chem*. 74 423–427. 10.1016/S0308-8146(01)00125-X

[B5] Asghari M. Rezaei-Rad R. (2018). 24-Epibrassinolide enhanced the quality parameters and phytochemical contents of table grape. *J. Appl. Bot. Food Qual*. 91 226–231. 10.5073/JABFQ.2018.091.030

[B6] Bajguz A. Chmur M. Gruszka D. (2020). Comprehensive overview of the brassinosteroid biosynthesis pathways: substrates, products, inhibitors, and connections. *Front. Plant Sci.* 11:1034. 10.3389/fpls.2020.01034 32733523 PMC7358554

[B7] Butelli E. Licciardello C. Zhang Y. Liu J. Mackay S. Bailey P. (2012). Retrotransposons control fruit-specific, cold-dependent accumulation of anthocyanins in blood oranges. *Plant Cell* 24 1242–1255. 10.1105/tpc.111.095232 22427337 PMC3336134

[B8] Carmona L. Alquézar B. Marques V. V. Peña L. (2017). Anthocyanin biosynthesis and accumulation in blood oranges during postharvest storage at different low temperatures. *Food Chem.* 237 7–14. 10.1016/j.foodchem.2017.05.076 28764055

[B9] Cebadera-Miranda L. Domínguez L. Dias M. I. Barros L. Ferreira I. C. F. R. Igual M. (2019). Sanguinello and Tarocco (*Citrus sinensis* [L.] Osbeck): bioactive compounds and colour appearance of blood oranges. *Food Chem*. 270 395–402. 10.1016/j.foodchem.2018.07.094 30174063

[B10] El-Otmani M. Ait-Oubahou A. Zacarías L. (2011). “*Citrus* spp.: orange, mandarin, tangerine, clementine, grapefruit, pomelo, lemon and lime,” in *Postharvest Biology and Technology of Tropical and Subtropical Fruits*, ed. Yahia E. M. (Cambridge: Woodhead Publishing), 437–516. 10.1533/9780857092762.437

[B11] Fallico B. Ballistreri B. Arena E. Brighina S. Rapisarda P. (2017). Bioactive compounds in blood oranges (*Citrus sinensis* (L.) Osbeck): level and intake. *Food Chem*. 215 67–75. 10.1016/j.foodchem.2016.07.142 27542451

[B12] Gao H. Kang L. N. Liu Q. Cheng N. Wang B. N. Cao W. (2015). Effect of 24-epibrassinolide treatment on the metabolism of eggplant fruits in relation to development of pulp browning under chilling stress. *J. Food Sci. Technol*. 52 3394–3401. 10.1007/s13197-014-1402-y 26028720 PMC4444923

[B13] Gao H. Zhang Z. K. Lv X. G. Cheng N. Peng B. Z. Cao W. (2016). Effect of 24-epibrassinolide on chilling injury of peach fruit in relation to phenolic and proline metabolisms. *Postharvest Biol. Technol*. 111 390–397. 10.1016/j.postharvbio.2015.07.031

[B14] Ghorbani B. Pakkish Z. (2014). Brassinosteroid enhances cold stress tolerance of Washington navel orange (*Citrus sinensis* L.) fruit by regulating antioxidant enzymes during storage. *Agric. Conspec. Sci.* 79 109–114.

[B15] Habibi F. Ramezanian A. (2017). Vacuum infiltration of putrescine enhances bioactive compounds and maintains quality of blood orange during cold storage. *Food Chem.* 227 1–8. 10.1016/j.foodchem.2017.01.057 28274408

[B16] Habibi F. Ramezanian A. Guillén F. Castillo S. Serrano M. Valero D. (2020a). Changes in bioactive compounds, antioxidant activity, and nutritional quality of blood orange cultivars at different storage temperatures. *Antioxidants* 9:1016. 10.3390/antiox9101016 33092024 PMC7589990

[B17] Habibi F. Ramezanian A. Guillén F. Serrano M. Valero D. (2020b). Blood oranges maintain bioactive compounds and nutritional quality by postharvest treatments with γ-aminobutyric acid, methyl jasmonate or methyl salicylate during cold storage. *Food Chem*. 306:125634. 10.1016/j.foodchem.2019.125634 31614291

[B18] Habibi F. Ramezanian A. Rahemi M. Eshghi S. Guillén F. Serrano M. (2019). Postharvest treatments with γ-aminobutyric acid, methyl jasmonate, or methyl salicylate enhance chilling tolerance of blood orange fruit at prolonged cold storage. *J. Sci. Food Agric*. 99 6408–6417. 10.1002/jsfa.9920 31283020

[B19] Hayat S. Ahmad A. (2011). *Brassinosteroids: A Class of Plant Hormone.* Dordrecht: Springer 10.1007/978-94007-0189-2

[B20] Huang R. H. Liu J. H. Lu Y. M. Xia R. X. (2008). Effect of salicylic acid on the antioxidant system in the pulp of ‘Cara Cara’ navel orange (*Citrus sinensis* L. Osbeck) at different storage temperatures. *Postharvest Biol. Technol*. 47 168–175. 10.1016/j.postharvbio.2007.06.018

[B21] Lado J. Rodrigo M. J. López-Climent M. Gómez-Cadenas A. Zacarías L. (2016). Implication of the antioxidant system in chilling injury tolerance in the red peel of grapefruit. *Postharvest Biol. Technol*. 111 214–223. 10.1016/j.postharvbio.2015.09.013

[B22] Lafuente M. T. Zacarías L. (2006). Postharvest physiological disorder in citrus fruit. *Stewart Postharvest Rev.* 2 1–9. 10.2212/spr.2006.1.2 25112557

[B23] Lafuente M. T. Establés-Ortiz B. González-Candelas L. (2017). Insights into the molecular events that regulate heat-induced chilling tolerance in citrus fruits. *Front. Plant Sci.* 8:1113. 10.3389/fpls.2017.01113 28694818 PMC5483458

[B24] Li B. Zhang C. Cao B. Qin G. Wang W. Tian S. (2012). Brassinolide enhances cold stress tolerance of fruit by regulating plasma membrane proteins and lipids. *Amino Acids* 43 2469–2480. 10.1007/s00726-012-1327-6 22660900

[B25] Lo Piero A. R. (2015). The state of the art in biosynthesis of anthocyanins and its regulation in pigmented sweet oranges (*Citrus sinensis* L. Osbeck). *J. Agric. Food Chem*. 63 4031–4041. 10.1021/acs.jafc.5b01123 25871434

[B26] Lo Piero A. R. Lo Cicero L. Puglisi I. (2014). The metabolic fate of citric acid as affected by cold storage in blood oranges. *J. Plant Biochem. Biotechnol.* 23 161–166. 10.1007/s13562-013-0197-7

[B27] Lo Piero A. R. Puglisi I. Rapisarda P. Petrone G. (2005). Anthocyanins accumulation and related gene expression in red orange fruit induced by low temperature storage. *J. Agric. Food Chem*. 53 9083–9088. 10.1021/jf051609s 16277406

[B28] MAPAMA (2019). *Ministerio de Agricultura y Pesca, Alimentación y Medio Ambiente.* Avaliable online at: http://www.mapama.gob.es/estadistica/temas/estadisticasagrarias/agricultura/default.aspx (accessed May 02, 2020)

[B29] Molinu M. G. Dore A. Palma A. D’Aquino S. Azara E. Rodov V. (2016). Effect of superatmospheric oxygen storage on the content of phytonutrients in ‘Sanguinello Comune’ blood orange. *Postharvest Biol. Technol*. 112 24–30. 10.1016/j.postharvbio.2015.09.037

[B30] Rapisarda P. Bellomo S. E. Intelisano S. (2001). Storage temperature effects on blood orange fruit quality. *J. Agric. Food Chem*. 49 3230–3235. 10.1021/jf010032l 11453756

[B31] Rezakhani M. S. Pakkish Z. (2017). Influences of brassinosteroid and hot water on postharvest enzyme activity and lipid peroxidation of lime (*Citrus aurantifolia* L.) fruit during storage at cold temperature. *Int. J. Hortic. Sci. Technol*. 4 57–65. 10.22059/IJHST.2017.207082.123

[B32] Rice-Evans C. A. Miller N. J. Paganga G. (1996). Structure–antioxidant activity relationships of flavonoids and phenolic acids. *Free Radic. Biol. Med*. 20 933–956. 10.1016/0891-5849(95)02227-9 8743980

[B33] Rivero R. M. Ruiz J. M. García P. C. López-Lefebre L. R. Sánchez E. Romero L. (2001). Resistance to cold and heat stress: accumulation of phenolic compounds in tomato and watermelon plants. *Plant Sci*. 160 315–321. 10.1016/S0168-9452(00)00395-2 11164603

[B34] Serrano M. Díaz-Mula H. M. Zapata P. J. Castillo S. Guillén F. Martínez-Romero D. (2009). Maturity stage at harvest determines the fruit quality and antioxidant potential after storage of sweet cherry cultivars. *J. Agric. Food Chem*. 57 3240–3246. 10.1021/jf803949k 19284725

[B35] Sharma S. Barman K. Prasad R. N. Sing J. (2020). “Chilling stress during postharvest storage of fruits and vegetables,” in *New Frontiers in Stress Management for Durable Agriculture*, eds Rakshit A. Singh H. B. Singh A. K. Sing U. S. Fraceto L. (Singapore: Springer Nature Singapore), 75–100. 10.1007/978-981-15-1322-0_6

[B36] Tomás-Barberán F. A. Espín J. C. (2001). Phenolic compounds and related enzymes as determinants of quality in fruits and vegetables. *J. Sci. Food Agric*. 81 853–876. 10.1002/jsfa.885

[B37] Valero D. Serrano M. (2010). *Postharvest Biology and Technology for Preserving Fruit Quality.* Boca Raton, FL: CRC Press 10.1201/9781439802670

[B38] Vardhini B. V. Rao S. S. R. (2002). Acceleration of ripening of tomato pericarp discs by brassinosteroids. *Phytochemistry* 61 843–847. 10.1016/S0031-9422(02)00223-6 12453577

[B39] Wang Q. Ding T. Gao L. Pang J. Yang N. (2012). Effect of brassinolide on chilling injury of green bell pepper in storage. *Sci. Hortic*. 144 195–200. 10.1016/j.scienta.2012.07.018

[B40] Wang Y. Luo Z. Huang X. Yang K. Gao S. Du R. (2014). Effect of exogenous γ-aminobutyric acid (GABA) treatment on chilling injury and antioxidant capacity in banana peel. *Sci. Hortic*. 168 132–137. 10.1016/j.scienta.2014.01.022

[B41] Wei Z. Li J. (2020). Regulation of brassinosteroid homeostasis in higher plants. *Front. Plant Sci* 11:1480. 10.3389/fpls.2020.583622 33133120 PMC7550685

[B42] Xu F. Xi Z. M. Zhang H. Zhang C. J. Zhang Z. W. (2015). Brassinosteroids are involved in controlling sugar unloading in *Vitis vinifera* ‘Cabernet Sauvignon’ berries during veraison. *Plant Physiol. Biochem*. 94 197–208. 10.1016/j.plaphy.2015.06.005 26113159

[B43] Zacarías L. Cronjé P. J. R. Palou L. (2020). “Postharvest technology of *Citrus* fruit,” in *The Genus Citrus*, eds Talón M. Caruso M. Gmitter F. G. (London: Woodhead Publishing), 421–446. 10.1016/B978-0-12-812163-4.00021-8

[B44] Zhu F. Yun Z. Ma Q. Gong Q. Zeng Y. Xu J. (2015). Effects of exogenous 24-epibrassinolide treatment on postharvest quality and resistance of Satsuma mandarin (*Citrus unshiu*). *Postharvest Biol. Technol*. 100 8–15. 10.1016/j.postharvbio.2014.09.01

[B45] Zhu T. Tan W. R. Deng X. G. Zheng T. Zhang D. W. Lin H. H. (2015). Effects of brassinosteroids on quality attributes and ethylene synthesis in postharvest tomato. fruit. *Postharvest Biol. Technol*. 100 196–204. 10.1016/j.postharvbio.2014.09.016

