# Supplementary figures and images for: Postharvest Application of 24-Epibrassinolide Reduces Chilling Injury Symptoms and Enhances Bioactive Compounds Content and Antioxidant Activity of Blood Orange Fruit
Source: Front Plant Sci. 2021 Feb 11;12:629733. doi: 10.3389/fpls.2021.629733 (PMC7905319; doi:10.3389/fpls.2021.629733)

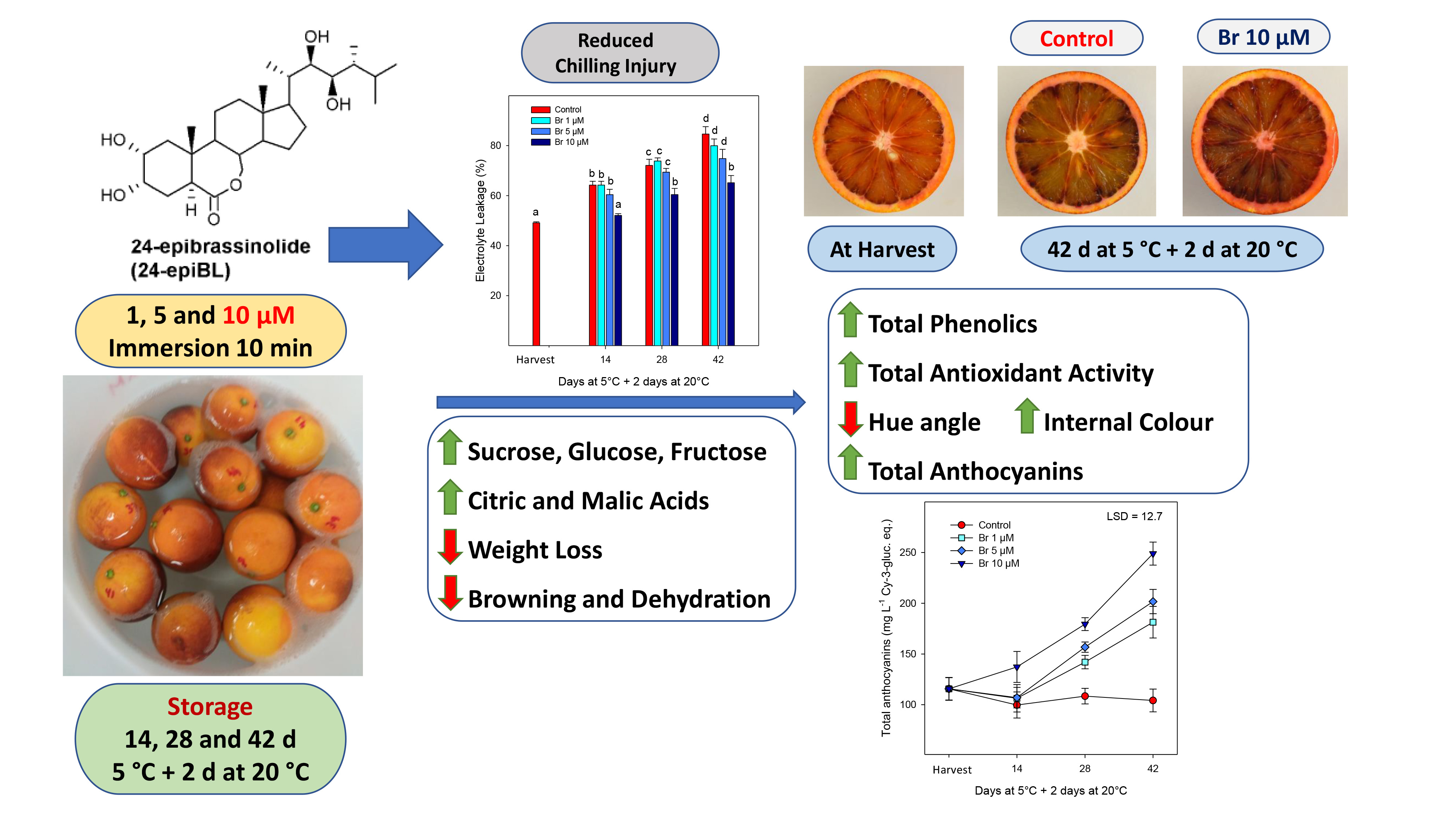

Supplement: Supplementary file 1 [file Image_1.JPEG]
